# In this month’s Bulletin 

**DOI:** 10.2471/BLT.23.000923

**Published:** 2023-09-01

In the editorial section, Mohamed El Amine Youcef Ali et al. (550) list the difficulties of addressing social determinants associated with cancer outcomes. Bruce Gordon et al. (551) describe the extent to which unsafe water, sanitation and hygiene persist as a public health problem. 

Tatum Anderson (554 – 555) reports on the use of new guidelines for the prevention and treatment of post-partum haemorrhage. Abla Mehio Sibai talks to Lynn Eaton (556 – 557) about cross-disciplinary approaches to healthy ageing and the challenges posed by the collapsing public sector in Lebanon.

## Angola, Bangladesh, Burkina Faso, Côte d’Ivoire, Ethiopia, Gambia, Ghana, India, Indonesia, Kenya, Nigeria, Pakistan, Papua New Guinea, Solomon Islands, South Africa, Türkiye, Uganda, United Republic of Tanzania, Viet Nam, Zambia

### Counting deaths due to cardiovascular disease 

Ajay Acharya et al. (571 – 586) review verbal autopsy studies for attributable mortality.

## Australia, India, Kenya, Nepal, New Zealand, United Kingdom of Great Britain and Northern Ireland 

### Regulation of health practitioners

Agya Mahat et al. (595 – 604) summarize recent reforms in six countries and discuss global implications. 

## Botswana, Burkina Faso, Burundi, Cameroon, Democratic Republic of Congo, Ethiopia, Ghana, Kenya, Malawi, Mozambique, Nigeria, Rwanda, Sierra Leone, Somalia, South Africa, Sudan, Uganda, United Republic of Tanzania, Zambia, Zimbabwe 

### Estimating chronic disease prevalence 

Stephen A Spencer et al. (558 – 570) review observational studies of hospitalized adults.

## Cambodia

### Tracking dengue 

Christina Yek et al. (605 – 616) summarize 18 years of national policies and surveillance data.

## India 

### Negotiating better prices for medicines

C S Pramesh et al. (587 – 594) report on a pilot initiative to pool procurement of antineoplastics.

